# Dysbiosis in the Dead: Human Postmortem Microbiome Beta-Dispersion as an Indicator of Manner and Cause of Death

**DOI:** 10.3389/fmicb.2020.555347

**Published:** 2020-09-04

**Authors:** Sierra F. Kaszubinski, Jennifer L. Pechal, Katelyn Smiles, Carl J. Schmidt, Heather R. Jordan, Mariah H. Meek, M. Eric Benbow

**Affiliations:** ^1^Department of Integrative Biology, Michigan State University, East Lansing, MI, United States; ^2^Department of Entomology, Michigan State University, East Lansing, MI, United States; ^3^Wayne County Medical Examiner’s Office, Detroit, MI, United States; ^4^Department of Pathology, University of Michigan, Ann Arbor, MI, United States; ^5^Department of Biological Sciences, Mississippi State University, Starkville, MS, United States; ^6^AgBio Research, Michigan State University, East Lansing, MI, United States; ^7^Ecology, Evolutionary Biology and Behavior Program, Michigan State University, East Lansing, MI, United States; ^8^Department of Osteopathic Medical Specialties, Michigan State University, East Lansing, MI, United States

**Keywords:** postmortem microbiome, forensic microbiology, manner of death, cause of death, microbial communities, beta-dispersion, Anna Karenina Principle, necrobiome

## Abstract

The postmortem microbiome plays an important functional role in host decomposition after death. Postmortem microbiome community successional patterns are specific to body site, with a significant shift in composition 48 h after death. While the postmortem microbiome has important forensic applications for postmortem interval estimation, it also has the potential to aid in manner of death (MOD) and cause of death (COD) determination as a reflection of antemortem health status. To further explore this association, we tested beta-dispersion, or the variability of microbiomes within the context of the “Anna Karenina Principle” (AKP). The foundational principle of AKP is that stressors affect microbiomes in unpredictable ways, which increases community beta-dispersion. We hypothesized that cases with identified M/CODs would have differential community beta-dispersion that reflected antemortem conditions, specifically that cardiovascular disease and/or natural deaths would have higher beta-dispersion compared to other deaths (e.g., accidents, drug-related deaths). Using a published microbiome data set of 188 postmortem cases (five body sites per case) collected during routine autopsy in Wayne County (Detroit), MI, we modeled beta-dispersion to test for M/COD associations *a priori*. Logistic regression models of beta-dispersion and case demographic data were used to classify M/COD. We demonstrated that beta-dispersion, along with case demographic data, could distinguish among M/COD – especially cardiovascular disease and drug related deaths, which were correctly classified in 79% of cases. Binary logistic regression models had higher correct classifications than multinomial logistic regression models, but changing the defined microbial community (e.g., full vs. non-core communities) used to calculate beta-dispersion overall did not improve model classification or M/COD. Furthermore, we tested our analytic approach on a case study that predicted suicides from other deaths, as well as distinguishing MOD (e.g., homicides vs. suicides) within COD (e.g., gunshot wound). We propose an analytical workflow that combines postmortem microbiome indicator taxa, beta-dispersion, and case demographic data for predicting MOD and COD classifications. Overall, we provide further evidence the postmortem microbiome is linked to the host’s antemortem health condition(s), while also demonstrating the potential utility of including beta-dispersion (a non-taxon dependent approach) coupled with case demographic data for death determination.

## Introduction

The organisms represented in microbiomes have important functional roles for host life – influencing health status, development, and disease susceptibility, among many others (Turnbaugh et al., 2007; Zaneveld et al., 2017). Microbes also play an important functional role in the decomposition process (Pechal et al., 2014), as the communities change with dispersal, competition, and other interactions after host death (Pechal et al., 2014, 2018; Metcalf et al., 2016). These dynamic, yet predictable (Belk et al., 2018; Pechal et al., 2018), microbial community profile changes after death make the postmortem microbiome a potential forensic resource for postmortem interval (PMI) estimation. PMI estimation is indeed the most studied forensic application of the postmortem microbiome (Metcalf et al., 2013; Pechal et al., 2014); but, this community has additional potential for other forensic applications as well, like indicating antemortem health conditions (e.g., cardiovascular disease or violent death) (Pechal et al., 2018) and the living environment (e.g., neighborhood blight) (Pearson et al., 2019).

The postmortem microbiome is structured in part by a decedents’ antemortem health condition and the suite of stressors that impact the human host. These stressors include drug/alcohol abuse or high stress lifestyle conditions like neighborhood blight (e.g., abandoned building, inactivity, and dumping), that are associated with certain manners of death (e.g., homicide) (Pechal et al., 2018; Pearson et al., 2019; Zhang et al., 2019). Importantly, the adult human postmortem microbiome does not significantly change from the antemortem microbiome for approximately 48 h after death when tested in a single geographic region (Pechal et al., 2018). Due to the stability of the postmortem microbiome within 48 h of death, and the potential connection to lifestyle condition, microbial community metrics (e.g., diversity) were associated with certain manners of deaths (MOD) or causes of deaths (COD) (Pechal et al., 2018; Zhang et al., 2019). However, fewer studies have tested associations of postmortem microbial community variability with MOD or COD (Pechal et al., 2018; Pearson et al., 2019; Zhang et al., 2019).

In past work, microbial diversity and indicator taxa were shown to reflect antemortem health conditions and MOD (Pechal et al., 2018; Pearson et al., 2019; Zhang et al., 2019). In some cases, lower microbial alpha-diversity was associated with cardiovascular disease, non-violent deaths, and neighborhood blight (Pechal et al., 2018; Pearson et al., 2019); however, it is difficult to capture the variability in a large sample set using alpha-diversity metrics alone (e.g., richness), as they do not account for taxon relative composition. Zhang et al. (2019) combined microbial indicator taxa and case demographic data found in autopsy reports (e.g., decedents age, sex, race, etc.) to test machine learning models for classifying M/COD (Zhang et al., 2019). While indicator taxa are a useful reflection of antemortem conditions, microbial indicator taxa may not be present in all cases (e.g., *Haemophilus influenzae*), or they may be ubiquitous (e.g., *Staphylococcus*), and so less useful for a generalizable tool for M/COD determination (Pechal et al., 2018). For this study, we tested a metric that captures microbial variability while not specifically relying on indicator taxa: beta-dispersion. Our goal was to determine how postmortem microbiome beta-dispersion could be an additional tool for predicting M/CODs during death investigation.

Following the conceptual context of the “Anna Karenina Principle” (AKP), after prolonged exposure to any array of stressors, the microbiomes of unhealthy individuals becomes more variable compared to the microbial communities of healthy individuals (Zaneveld et al., 2017). For example, beta-dispersion increased among living individuals with a history of obesity, infection, and smoking (Barbian et al., 2015; Zaneveld et al., 2017). In other words, increased variation in the microbial communities reflects dysbiosis, and this community variability can be quantified through calculations of beta-dispersion (Barbian et al., 2015; Zaneveld et al., 2017). Beta-dispersion is calculated, within the context of a dataset, using a multivariate distance from the centroid for each case sample, as defined by a grouping factor (e.g., body site, PMI, weight status) (Oksanen et al., 2019). Based on the link between increased beta-dispersion and health status in living populations, we considered M/COD as grouping factors to quantify microbial signatures and develop metrics associated with M/COD determinations. Such association of beta-dispersion with M/COD could conceivably be additional evidence in future death investigation.

Microbial community metrics could potentially aid medical examiners and other certifiers of death (referred to as “medical examiners”), as determining M/COD can be error prone. While COD spans a variety of causes relating to the injury/disease a person died from, MOD encompasses only five major categories: natural, accident, suicide, homicide, and indeterminate (Randy et al., 2002). Medical examiners qualify their MOD determination with incremental degrees of certainty considering the available evidence (Randy et al., 2002). Given the possibility for mismatch between the MOD determination and the actual MOD, the postmortem microbiome could provide another potential piece of evidence to document M/COD determination.

To evaluate how postmortem microbiome variability is associated with M/CODs, we modeled postmortem microbiome beta-dispersion from five body sites of 188 routine autopsy with known M/COD (as determined by a board-certified forensic pathologist). We predicted that certain M/CODs, such as natural deaths and cardiovascular disease, would have higher beta-dispersion than other M/CODs due to the previous antemortem health condition links found in previous studies (Pechal et al., 2018). However, the effect of life environment was predicted to increase beta-dispersion as well, in a way that could potentially factor into deaths classified as homicide, for example, those due to blunt force trauma or gunshot wounds (Pearson et al., 2019). Quantifying beta-dispersion, using M/COD as a grouping factor, could provide reliable and usable tool in death investigation for M/COD determination.

## Materials and Methods

### Sample Collection, DNA Extraction, and Sequencing

The postmortem microbiome data used in this study were acquired from Pechal et al. (2018) but re-analyzed to test M/COD determination from beta-dispersion. This dataset contains postmortem microbiome samples obtained from 188 Wayne County Medical Examiner’s Office autopsy cases (Detroit, MI, 2014–2016), representing multiple MODs (accident, homicide, suicide, natural) and CODs (asphyxiation, blunt force trauma, cardiovascular disease, drug-related deaths, gunshot wounds, etc.) (Supplementary Table S1) (Pechal et al., 2018). The cases also represent a cross-section of the greater Detroit metropolitan area population and were nearly evenly divided among females and males (83:105) and black and white (90:98) (Supplementary Table S1). Cases comprised of adults (18–88 years) with a body mass index (BMI) ranging from 8.5–67.5 kg/m^2^ (Supplementary Table S1). The dataset is the largest postmortem microbiome available to test beta-dispersions potential to aid in M/COD determination.

Detailed methods for sample collection, DNA extraction, and sequencing can be found in Pechal et al. (2018). To summarize, trained personnel at Wayne County Medical Examiner’s Office collected microbial community swab samples from five body sites (nose, mouth, rectum, ears, and eyes) during routine autopsy. Microbial DNA was extracted and sequenced to characterize the microbial communities. The Michigan State University (MSU) Genomics Core Facility (East Lansing, MI, United States) sequenced the 16S rRNA V4 region using an Illumina MiSeq standard flow cell (v2) using a 500-cycle reagent cartridge.

### Data Analysis and Bioinformatics

Sequence reads from postmortem microbiome samples were analyzed with QIIME2 (v2018.11) (Bolyen et al., 2019), following the methodology outlined in Kaszubinski et al. (2019). DADA2 v1.8.0 (Callahan et al., 2016) was used for denoising. Sequences were aligned using MAFFT v7.397 (Katoh and Standley, 2013), and FastTree v2.1 (Price et al., 2010). Taxonomy and amplicon sequencing variant (ASV) tables were exported as comma separated values (csv) files to be used as input data for all downstream analysis. ASV and taxonomy files were combined with demographic data obtained from autopsy reports (age, sex, race, BMI, etc.) as *phyloseq* (v1.28.0) objects in R (v3.6.1) (McMurdie and Holmes, 2013; R Core Team, 2018). ASVs less than 0.01% of the mean library size were trimmed, removing 22,214 ASVs for a total of 8,692 ASVs. *Phyloseq* objects were split among the body sites (nose, mouth, rectum, ears, and eyes) and analyzed separately.

#### Method Selection

To determine the optimal methodology for calculating beta-dispersion before moving forward with classifying M/COD, we compared standardization approaches of the microbial communities, distance matrices, and alpha-diversity to select the optimal method for beta-dispersion calculation. For standardization, rarefying (randomly subsampling ASVs to a specified minimum library size) and normalizing (non-rarefaction based method; removing ASVs not present in a specified percentage of samples) were compared for each body site using three minimum library sizes (3,000, 5,000, and 7,000 sequences) and sample percentage cut off (1%, 3%, 10%), respectively. We wanted to determine not only which standardization strategy was optimal, but also how sensitive the standardizations were (minimum library size and sample percentage cut off). While rarefaction has been debated (McMurdie and Holmes, 2014; Weiss et al., 2017), we sought to eliminate bias associated with different library sizes that could inflate differences in beta-dispersion among M/CODs (Kaszubinski et al., 2019). Specifically, library size differences can mask biologically meaningful results especially for unweighted distance matrices (Weiss et al., 2017). Normalization (as referenced in this manuscript), is a common non-rarefaction technique used in ecology studies (Poos and Jackson, 2012). However, this leads to different library sizes among samples, which may be an artifact in some analyses.

We also compared unweighted and weighted UniFrac distances matrices for calculating beta-dispersion to determine whether considering abundances (weighted UniFrac) would affect the beta-dispersion calculation and should be considered for downstream modeling; UniFrac (weighted and unweighted) is commonly used in forensic studies (Javan et al., 2017; Pechal et al., 2018). Beta-dispersion was calculated among MODs and CODs using the *vegan* package (v2.5-5) in R at each minimum library size and sample percentage cutoff (Oksanen et al., 2019). The *betadisper* function from vegan reports the distance from the centroid for each sample, as defined by a grouping factor (in this dataset M/COD). Each postmortem microbiome sample had two corresponding beta-dispersion values, with either MOD or COD as a grouping factor. Kruskal–Wallis, Fligner-Killeen, and *post hoc* Nemenyi tests among beta-dispersion values tested differences among M/CODs and were reported with a Bonferroni correction (Pohlert, 2018). Additionally, alpha-diversity metrics (Chao1 and Shannon diversity) were calculated using *phyloseq* for each minimum library size and sample percentage cutoff level.

We then selected a methodology [standardization of input data analysis (rarefaction or normalization) and distance matrix] for calculating beta-dispersion based on the number of significant differences in beta-dispersion (among M/CODs) identified by Kruskal–Wallis and *post hoc* Nemenyi tests as well as the highest alpha-diversity (see the section “Method Selection” in “Results”). This standardization approach was identified for its potential to distinguish M/COD, while maintaining microbial diversity. For subsequent analyses, microbial communities were rarefied to 5,000 sequences and beta-dispersion was calculated using unweighted UniFrac distance.

#### Model Selection

We built multinomial logistic regression models to classify M/COD from beta-dispersion values and case demographic data (Böhning, 1992) using the *lme4* (v1.1-21) and *mlogit* package (v1.0-1) (Bates et al., 2019; Croissant, 2019). Logistic regression is an analysis commonly used in clinical settings because it has distinct advantages (de Jong et al., 2019). Logistic regression does not assume normality, linearity, or homoscedasticity (even variance) (Harrell et al., 1996; Steyerberg et al., 2001). Logistic regression is prone to overfitting, especially in cases with small datasets (de Jong et al., 2019). However, goodness-of-fit metrics, such as R^2^ and model comparison can be used to evaluate the validity of models (Pohlert, 2018; de Jong et al., 2019).

Full multinomial logistic regression models included all categories of interest for classifying M/COD (e.g., homicide, suicide, natural, and accidental death for MOD; cardiovascular disease, drug related deaths, blunt force trauma, asphyxiation, gunshot wounds, and “other deaths” for COD). Demographic data of interest [age, BMI, sex, race, PMI (<48 h; >49 h), season, and event location (outdoors, indoors, hospital, vehicular)] were summarized (Table 1) and demographic means were tested among M/CODs (Kruskal–Wallis with a Bonferroni correction) to identify potential significant (*p* < 0.05) covariates for inclusion in model building (Pohlert, 2018). Multicollinearity was evaluated among the covariates using a correlation test, which found the covariates to be independent, meeting model assumptions (Supplementary Figure S1) (Steyerberg et al., 2001).

**TABLE 1:** Case demographic data stratified by M/COD.

	Age (y)		BMI		Sex		Race		PMI		Event location				Season			
	Mean	SD	Mean	SD	Female	Male	Black	White	<48 h	>49 h	Hospital	Indoors	Outdoors	Vehicular	Autumn	Spring	Summer	Winter
**Manner of death**																		
Accident (*n* = 71)	39.9	13.8	30	8.48	35	36	26	45	62	9	10	51	7	3	4	41	17	9
Homicide (*n* = 39)	36.2	12.3	26.9	6.42	9	30	34	5	37	2	9	14	12	4	2	20	6	11
Natural (*n* = 57)	53.7	10.7	29.8	10.1	25	32	29	28	45	12	6	49	1	1	2	33	14	8
Suicide (*n* = 22)	44.9	15.6	27.2	6.57	11	12	3	20	21	2	1	18	3	1	2	10	6	5
**Cause of death**																		
Asphyxiation (*n* = 11)	46.5	20.1	25.4	4.35	4	7	2	9	10	1	1	7	2	1	1	3	3	4
Blunt force trauma (*n* = 21)	42.6	14.7	28.9	10.3	12	9	14	7	21	0	4	7	8	2	4	9	3	5
Cardiovascular disease (*n* = 42)	54.5	11.6	29.9	9.56	20	22	23	19	34	8	6	35	1	0	2	26	7	7
Drug-related deaths (*n* = 70)	41	12.7	29.7	7.51	34	36	22	48	58	12	5	60	3	2	2	43	17	8
Gunshot wounds (*n* = 30)	33.8	11.8	27.5	6.34	5	25	24	6	28	2	7	10	9	4	1	16	6	7
Other (*n* = 16)	47.3	12	28.8	12.1	5	11	7	9	14	2	3	13	0	0	0	7	7	2

*Mean (±SD) age (years) and body mass index (BMI; kg/m^2^) for 188 cases included in this study. Sex, race, postmortem interval (PMI) estimate, death event location, and time of year (season) for death discovery were reported as count data.*

Multiple logistic regression models classifying M/COD were built and tested for each body site. We used a stepwise selection with backward elimination of predictors to determine significant covariates (Steyerberg et al., 2001). Only those models with significant (*p* < 0.1) beta-dispersion contribution as a covariate were selected for further modeling with case demographic data. We chose a more conservative p-value for the beta-dispersion covariate cut off to avoid excluding potentially relevant models for further evaluation, as recommended in other studies (Steyerberg et al., 2001). However, overall model significance was considered at *p* < 0.05. The best performing models were identified based on goodness-of-fit metric McFadden *R*^2^ (excellent fit ranges from 0.2-0.4) (McFadden, 1979), model comparison using Akaike information criterion (AIC) (lower AIC = better model) (Bozdogan, 1987), and classification success (correct classifications/total number of samples). Based on these initial model building results, models could be improved (see the section “Model Selection” in “Results”).

To improve logistic regression models, we considered three microbial community types for beta-dispersion calculation: full communities, random forest indicator communities, and “non-core” communities. While the grouping factor (M/COD) remained the same, the microbial communities used to calculate beta-dispersion differed. “Full community” beta-dispersion was calculated from the standardized community input data within each body site. “Random forest indicators,” as determined by *Boruta* (v6.0.0) in R, used confirmed and tentative taxa of importance (*p* < 0.05) (Kursa and Rudnicki, 2018), and beta-dispersion was calculated from this set of significant indicator taxa. Random forest classification error was determined using the *randomForest* package (v4.6-14) in R (Breiman et al., 2018). While the definition of a “core microbiome” is widely debated (Shade and Handelsman, 2012), we defined “non-core” taxa in this study as taxa present in only one M/COD. The taxa had to be present in at least one case, for example the genus *Turicella* was present in only one homicide case, while the family Streptococcaceae was represented in several homicide cases. “Core” taxa were removed, and beta-dispersion was calculated from the remaining ‘non-core’ taxa.

Lastly, we tested binary logistic regression (classifying between two categories) compared to multinomial logistic regression (multiple categories). Binary logistic regression models were also built using the *lme4* (v1.1-21) and *mlogit package* (v1.0-1) in R (Bates et al., 2019; Croissant, 2019), and the best performing models were considered based on AIC (Bozdogan, 1987), McFadden *R*^2^, and classification success (correct classifications/total number of samples). Beta-dispersion differences were visualized using principal coordinate analysis (PCoA) plots created in *phyloseq*, and potential beta-dispersion differences were assessed using permutational multivariate analysis of variance (PERMANOVA) (Anderson, 2017).

#### Case Studies

Using the methodology outlined above, we tested data from two case studies for classifying MOD from nose communities to showcase the forensic potential beta-dispersion has as a tool for medical examiners. For the first case study (Case Study #1), a matched design with paired cases of similar age, race, and sex, to limit the effect of demographic data (Supplementary Table S2), were examined to compare suicides (*n* = 22) against other manners of death (*n* = 21 accident, homicide, or natural). For the second case study (Case Study #2), we examined MOD within COD, specifically examining homicides (*n* = 25) vs. suicides (*n* = 4) resulting from gunshot wounds (Supplementary Table S1). Potential indicator taxa for each grouping (MOD) were identified using *Boruta* in R. We also evaluated the potential beta-dispersion differences between MODs using PERMANOVA (Anderson, 2017), and classified MOD using binary logistic regression. We calculated achieved power using *G^∗^Power 3* v3.0.5 (Faul et al., 2007). Case study beta-dispersion was compared using the mean and standard deviation using an independent mean two−tailed *t*-test (α = 0.05).

## Results

### Method Selection

Unweighted UniFrac distances, compared to weighted UniFrac distances, were the optimum distance matrix for these data. In summary, unweighted UniFrac had three-times as many significant comparisons (identified mean differences of beta-dispersion among M/CODs by Kruskal–Wallis and *post hoc* Nemenyi *p* < 0.05) across rarefied and normalized communities, four-times as fewer deviations of variance (identified variance differences of beta-dispersion among M/CODs by Fligner-Killeen *p* < 0.05), and lack of library size bias (Supplementary Figures S2, S3 and Supplementary Tables S3, S4). Unifrac distances were more robust against rarefying and normalizing, as significant comparisons occurred with both standardization methods (Kruskal–Wallis and *post hoc* Nemenyi *p* < 0.05; Supplementary Figure S3 and Supplementary Table S3). Rarefaction was the more appropriate standardization strategy than normalization for this dataset as well. While normalizing the data had more than double the significant comparisons than rarefying, normalizing microbial communities led to a significant decrease in alpha-diversity compared to rarefying (Kruskal–Wallis and *post hoc* Nemenyi *p* < 0.05; Supplementary Figures S2, S4 and Supplementary Tables S3, S5). For normalizing combined with Weighted Unifrac, we found a bias of library size among the significant comparisons (Supplementary Table S4). Most of the significant comparisons (7 out of 10) had differential library sizes, reflecting that M/COD differences in beta-dispersion may have been related to library size using our normalization approach (Kruskal–Wallis and *post hoc* Nemenyi *p* < 0.05; Supplementary Table S4). A minimum library size of 5,000 sequences was selected for model comparisons, as more body sites yielded significant comparisons (Kruskal–Wallis *p* < 0.05) than other library sizes (7,000: 3 body sites, 5,000: 5 body sites; 3,000: 1 body site; Supplementary Figure S2) and was the appropriate minimum library size based on alpha-rarefaction curves of sequencing depth (Supplementary Tables S3, S5 and Supplementary Figures S5, S6).

### Model Comparison

**FIGURE 1:**
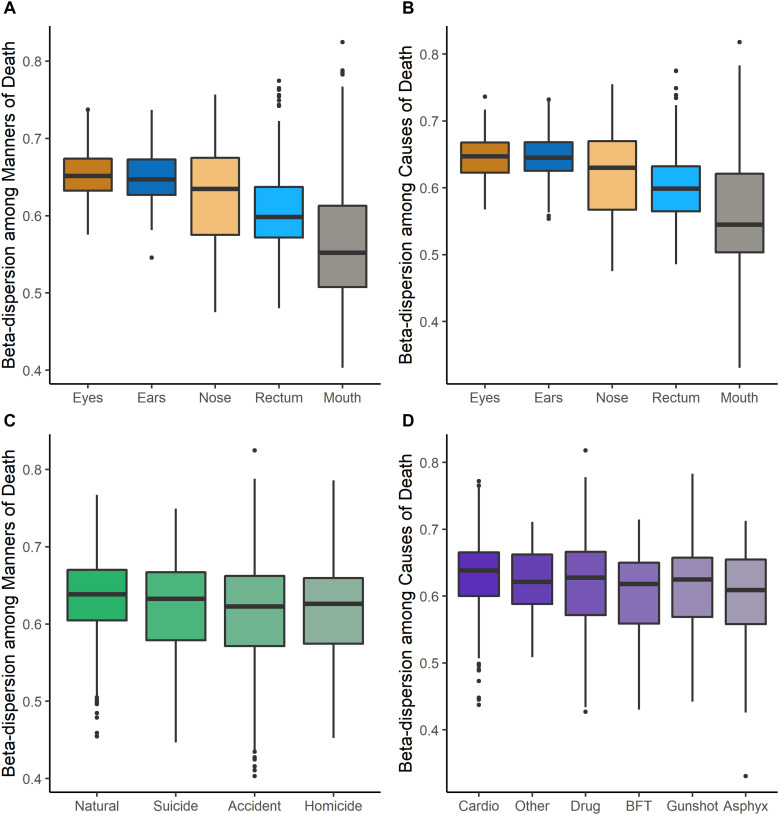
Beta-dispersion values (distances from the centroid) for each postmortem sample, stratified among body sites and MODs/CODs. **(A)** Beta-dispersion values with MOD as the grouping factor and stratified among body sites. **(B)** COD as the grouping factor and stratified among body sites. **(C)** Beta-dispersion values for all postmortem samples with MOD as the grouping factor, **(D)** with COD as the grouping factor (cardio, cardiovascular disease; drug, drug-related deaths; BFT, blunt force trauma; asphyx, asphyxiation).

Beta-dispersion significantly differed among body sites and M/CODs (Kruskal–Wallis *p* < 0.05, Figure 1 and Supplementary Table S6). Every postmortem microbiome sample had two corresponding beta-dispersion values (distances from centroid), with either MOD or COD as the grouping factor. On average, eye microbiomes had the highest beta-dispersion [MOD: 0.646 (SD = 0.0346); COD: 0.642 (SD = 0.0346); Supplementary Table S6], while mouth communities had the lowest beta-dispersion [MOD: 0.567 (SD = 0.0779); COD: 0.563 (SD = 0.0800); Supplementary Table S6]. Beta-dispersion was significantly different among all body site communities, except the ears and eyes, but we considered all body sites for downstream modeling with logistic regression to not prematurely remove body sites from consideration (Kruskal–Wallis *p* < 0.05; Supplementary Table S6). Natural death postmortem microbiomes had the highest average beta-dispersion [0.628 (SD = 0.0560); Supplementary Table S6] compared to homicides [0.606 (SD = 0.0694)] and accidents [0.608 (SD = 0.0683); Kruskal–Wallis *p* < 0.05; Supplementary Table S6]. Microbiomes of cases with cardiovascular disease had significantly higher beta-dispersion among all body sites [0.625 (SD = 0.0565); Supplementary Table S6] compared to gunshot wounds [0.605 (SD = 0.0708)], blunt force trauma [0.601 (SD = 0.0624)] and drug-related deaths [0.611 (SD = 0.0684); Kruskal–Wallis *p* < 0.1; Supplementary Table S6]. While beta-dispersion means differed significantly among MODs and CODs, there was overlap among beta-dispersion values, indicating that other variables contribute to microbiome beta-dispersion (Figure 1). Therefore, we considered additional case demographic data for downstream modeling. Age, sex, race, and event location were significantly different among MODs/CODs (Kruskal–Wallis *p* < 0.05; Table 1; Supplementary Table S7); however, so as to not prematurely remove potentially important demographic data we included all demographic data of interest in downstream modeling [age, BMI, sex, race, PMI (<48 h; >49 h), season, and event location (outdoors, indoors, hospital, vehicular)].

Multinomial logistic regression models were useful for initially determining which body site beta-dispersion had the best classification potential for M/COD. Classifying among all MODs, nose and mouth beta-dispersion were significant covariates, while nose, mouth, and ear beta-dispersion were a significant covariate for classifying all COD categories (*p* < 0.1; Supplementary Table S8). Nose community beta-dispersion, on average, successfully classified MODs at 61.1% (SD = 0.872), while ears and nose beta-dispersion successfully classified CODs on average 62.3% (SD = 0.599) and 62.5% (SD = 0.291), respectively (Supplementary Table S8). Based on these results, we only used those body sites with statistically significant (*p* < 0.05) models among M/CODs for further model building: nose, mouth, and ears.

While initial multinomial logistic regression models were able to classify among all M/CODs at higher success rate than random (overall average 50.2%; random chance: MOD: 25.0%; COD: 16.7%), models could be improved (Supplementary Table S8). Models classifying M/COD with only beta-dispersion (no case demographic data) were significant (*p* < 0.05) but had low classification success and model fit (∼40% average classification success; McFadden *R*^2^ of ∼ 0.0244; Supplementary Table S8). Adding case demographic data to the models led to better classification success and model fit (∼60% average classification success; McFadden *R*^2^: ∼0.298; Supplementary Table S8). However, we attempted to improve our models by testing different microbial communities to calculate beta-dispersion (full communities, random forest indicator communities, and “non-core” communities) and binary logistic regression rather than multinomial logistic regression.

**FIGURE 2:**
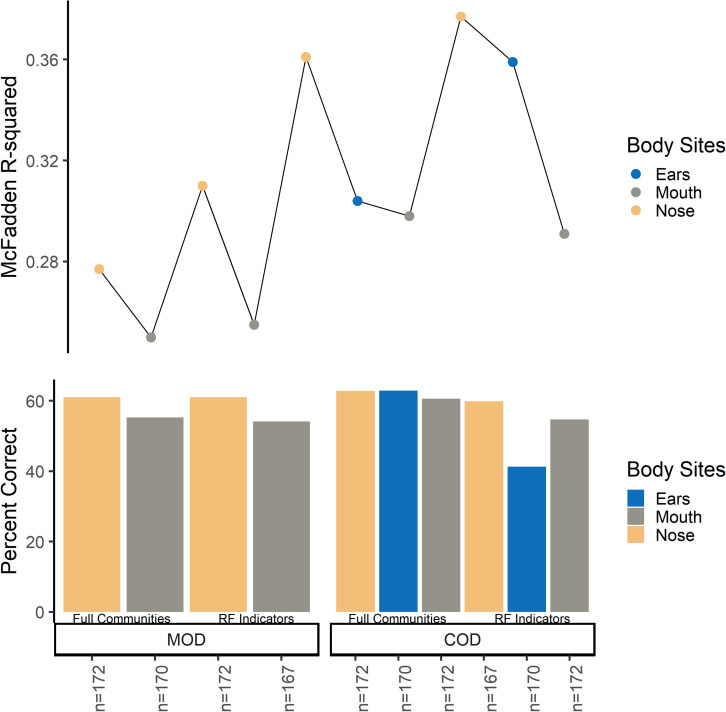
Multinomial logistic regression model comparison among full communities and random forest indicator communities beta-dispersion for MODs/CODs. For the bottom panel, the y-axis indicates percent correct, or the number of correct classifications/total number of samples. Each bar represents a multinomial logistic model. For the top panel, the y axis indicates McFadden *R*^2^ for the corresponding multinomial logistic regression model.

Overall, microbial community type (full communities, random forest indicator communities, and “non-core” communities) did not improve logistic regression models (Figure 2 and Supplementary Tables S9, S10). Models using “non-core” community beta-dispersion were not significant (*p* > 0.05) and so thus removed from further consideration and downstream modeling (Supplementary Table S9). Even though random forest indicator communities were specific to M/COD, multinomial logistic regression models were not more successful than full communities at classifying M/COD, and were less successful in some cases [percent correct classifications for full: 59.1% (SD = 2.64); RF: 57.8% (SD = 3.06); Figure 2; Supplementary Table S10]. For the random forest indicator communities and full communities, all models were within 7% of each other for McFadden *R*^2^, a metric of model fit [full: 0.298 (SD = 0.0367); RF: 0.318 (SD = 0.0445); Figure 2; Supplementary Table S10].

**FIGURE 3:**
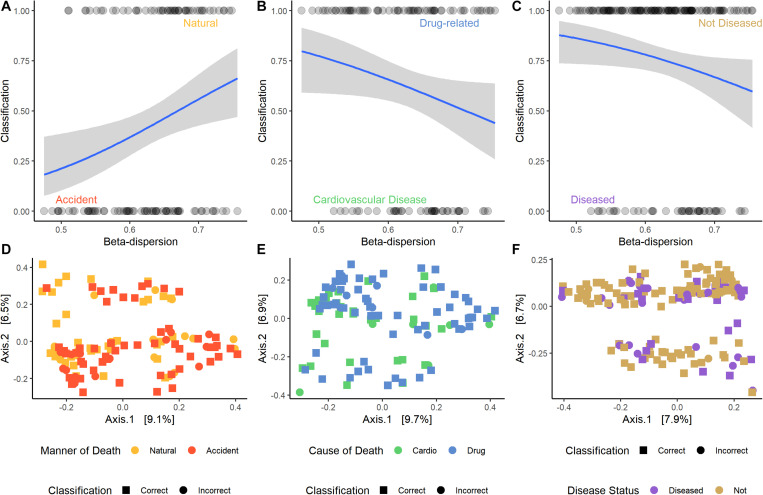
Logistic regression models of the best performing pair-wise comparisons. Nose samples were selected, as well as beta-dispersion from full communities. **(A–C)** Logistic regression models with a 95% confidence interval of beta-dispersion on the *x*-axis and the binary classification on the *y*-axis. **(A)** Accident: 0, natural: 1. **(B)** Cardiovascular disease: 0, drug-related: 1. **(C)** Disease: 0, non-diseased: 1. **(D–F)** Principal coordinate analysis (PCoA) plots of microbial samples included in the logistic regression model. Colors correspond with the M/COD, while shape indicates if the sample was correctly classified by the model. **(D)** Natural vs. accidental deaths. **(E)** Drug-related vs. cardiovascular disease deaths. **(F)** Diseased vs. non-diseased deaths.

For some M/COD comparisons (natural vs. accidental death; cardiovascular disease vs. drug-related death; disease vs. non-diseased state), binary logistic regression models performed best with an average classification success of 83.2% (SD = 5.50) (Supplementary Table S11) for full community and random forest indicator communities (“non-core” communities were not modeled for binary logistic regression due to poor results in the multinomial logistic regression models). For nose communities, beta-dispersion was a significant covariate (*p* < 0.1) between natural vs. accidental death, cardiovascular disease vs. drug-use, and diseased (natural deaths) vs. non-diseased (accidental, homicide, suicide) deaths (Table 2 and Supplementary Table S11). While random forest indicator communities had marginally higher successful classification compared to full communities (full: 78.9%; RF: 83.6%) and higher McFadden *R*^2^ (full: 0.347; RF: 0.369), the sample size of cases included in the random forest models was smaller (Table 2 and Supplementary Table S11), as some samples were discarded if they did not have the RF indicator taxa. Therefore, we considered full community beta-dispersion as the most appropriate metric (Figure 3). There was no distinct visual clustering of samples suggesting that misclassification was randomly distributed among samples (PERMANOVA *p* > 0.05; Figure 3). In summary, we have compiled the best performing multinomial and binary logistic regression models for this dataset, based on the percent correct classifications, McFadden *R*^2^, and AIC (Supplementary Table S12).

**TABLE 2:** Summary of binary logistic regression models classifying natural vs. accident, cardiovascular vs. drug-use, and disease vs. non-diseased state.

Comparison	Beta-dispersion community profile	Significant demographic data	McFadden *R*^2^	Degrees of freedom	χ^2^	*P*	Accuracy
Natural – Accident	Full community (*n* = 120)	Race + event location + age	0.314	6	51.9	<< 0.05	0.783
	Random forest indicators (*n* = 117)	Event location + age	0.388	5	62.5	<< 0.05	0.829
Cardio – Drug	Full community (*n* = 107)	BMI + event location + PMI + age	0.399	5	56.9	<< 0.05	0.804
	Random forest indicators (*n* = 100)	BMI + age	0.356	3	47.6	<< 0.05	0.820
Disease – Non	Full community (*n* = 172)	BMI + race + event location + age	0.328	5	70.3	<< 0.05	0.779
	Random forest indicators (*n* = 163)	BMI + race + event location + age	0.364	7	73.1	<< 0.05	0.859

*Significance of the model was determined by the *p*. For model comparisons, the McFadden *R*^2^ and model accuracy (correct classifications/total number of samples) were considered within each body site.*

### Case Studies

#### Case Study #1

**FIGURE 4:**
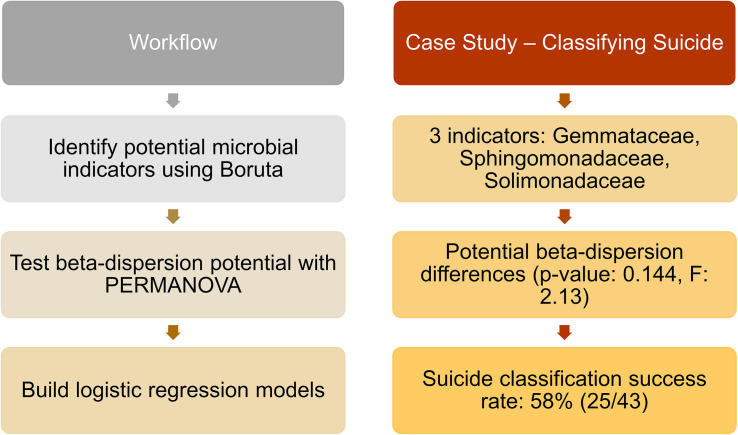
Proposed workflow with suicide matched-design case study. **Left** column indicates potential steps researchers and practitioners can follow for future studies. **Right** column provides results from the matched-design case study, following the workflow. Twenty-three suicide cases were matched by age, sex, and race with other deaths (natural, homicide, and accidental), and nose samples were included in the analyses. Indicator taxa were identified by *Boruta*, while beta-dispersion was calculated using UniFrac distances and tested with PERMANOVA. A logistic regression model of beta-dispersion was constructed to classify suicide vs. non-suicide deaths.

Of the 188 cases, we matched nose communities of 22 cases by age [43 years (SD = 14)], sex (21 females; 22 males), and race (6 blacks; 37 whites) with other deaths (natural, homicide, and accidental) for a total of 43 cases (Supplementary Table S2). We identified three significant indicator taxa of suicide (*Boruta p* < 0.05; Supplementary Table S13). Suicide communities had higher beta-dispersion [0.659 (SD = 0.0433)] than non-suicides [0.654 (SD = 0.0427); Supplementary Table S13] but there were no significant differences in beta-dispersion among suicides (PERMANOVA permuted *p* = 0.144; Supplementary Table S13). Logistic regression of beta-dispersion without demographic data classified suicide cases with a 58.1% success rate (Supplementary Table S13), likely associated with low power (1–β: 0.0660). For future studies, we proposed a potential workflow using this matched-design case study for other researchers to use as a reference (Figure 4 and Supplementary Table S13).

#### Case Study #2

Despite the low sample sizes, we identified ten potential indicator taxa for homicide vs. suicide resulting from gunshot wounds (*Boruta p* < 0.05; Supplementary Table S14). Beta-dispersion among gunshot wound homicides was significantly higher [0.626 (SD = 0.0491)] than gunshot wound suicides [0.543 (SD = 0.0959); PERMANOVA permuted *p* < 0.05; Supplementary Table S14]. Furthermore, we found significant logistic regression models of homicides vs. suicides with gunshot wounds accurately classified 93.1% of the time (Supplementary Table S14), despite uneven and low sample sizes (*n* = 25 homicides; *n* = 4 suicides; Supplementary Table S14), we achieved moderate power (1–β: 0.469).

## Discussion

In previous research, the AKP concept was tested with distinct treatment vs. control groups based on living health conditions and in living hosts (Zaneveld et al., 2017). However, we postulated the postmortem microbiome of various M/CODs would correspond with differential beta-dispersion, which could potentially be used as additional evidence in future forensic investigations. We hypothesized that cardiovascular disease and/or natural deaths would have the highest beta-dispersion, as AKP correlates with disease state in the antemortem life condition (Barbian et al., 2015; Zaneveld et al., 2017). Higher microbiome beta-dispersion was also predicted to be related to a stressful life environment, which is often associated with homicides, gunshot wounds, and blunt force trauma deaths (Pearson et al., 2019). Our best performing models were binary logistic regression models that confirmed the medical examiner’s M/COD assessment ∼79% of the time, specifically for cardiovascular disease vs. drug-related deaths. Multinomial logistic regression models confirmed the medical examiner’s M/COD assessment nearly 62% of the time. While better than random chance, including all M/CODs during classification (with uneven sample sizes) likely resulted in reduced classification accuracy for the multinomial logistic regression models.

Our dataset represents a cross-section of deaths from a large metropolitan area, with multiple body sites, using targeted sequencing of the 16S rRNA gene. The cases included were predominately natural cardiovascular disease deaths and accidental drug-related deaths. Therefore, direct comparison of the results of this study would be most applicable for cities with similar demographics (U.S. Census Bureau, 2020a), such as Chicago, IL (U.S. Census Bureau, 2020b) or Cincinnati, OH (U.S. Census Bureau, 2020c). While the demographic data lends classification ability in multinomial logistic regression, areas with differing demographics will require the creation of independent baseline models. It is also important to note that beta-dispersion is calculated in reference to the samples included in the dataset (Anderson et al., 2006). Therefore, future work should include data and cases from multiple geographic areas that include a range of socio-economic diversity and overall living conditions.

While we included five body site (ears, nose, mouth, eyes, and rectum) communities that showed differential success in classifying M/COD with beta-dispersion, there are more body sites of interest for the forensic community. As body site drives the microbial community composition more than any other factor (Pechal et al., 2018), comparisons to other body sites may be limited. For example, body sites sampled for the internal organs and blood (Javan et al., 2016) or skin microbiome (Kodama et al., 2019) could harbor different microbial communities than the ones included in this study, and provide different predictive power using this modeling approach. Beta-dispersion among all body sites was significantly different, but mouth, nose, and ears from this data set showed the most potential for downstream forensic applications. This is due to beta-dispersion from these body sites being significant covariates in logistic regression models, but also because these body sites are exposed to the environment and can potentially be affected by ambient conditions (Dewhirst et al., 2010). Dysbiosis of the oral cavity (nose, mouth, ears) has also been linked to systemic diseases such as cardiovascular disease (Seymour et al., 2007), and could link to the results we report here with higher beta-dispersion in cardiovascular disease and natural deaths.

This work revealed that beta-dispersion has potential to inform the M/COD decision making process during death determination. Accidental deaths, which were predominately drug related deaths in this dataset, had overall lower beta-dispersion than natural deaths, mirroring the dysbiosis found in non-forensic studies (Meckel and Kiraly, 2019). Accidental deaths and homicides were not distinguishable by beta-dispersion. While we hypothesized that high-stress lifestyle associated with homicidal deaths would increase beta-dispersion (Pearson et al., 2019), homicides had the lowest beta-dispersion among MODs. The antemortem link of high-stress lifestyle was not as strong as antemortem disease status in this study, compared to previous results that indicated higher microbial diversity associated with neighborhood blight and vacancy (Pearson et al., 2019). This may be because those decedents who were victims of homicide lived relatively healthy lifestyles and were, overall, younger when compared to decedents with a disease status. However, we do not have access to that specific information, as we were constrained to the contents of the autopsy reports.

In suicides, postmortem microbiomes of the nose, while representing the lowest sample size, had similar beta-dispersion to natural deaths, which was similar to other antemortem studies (Naseribafrouei et al., 2014; Liang et al., 2018). Microbiomes of suicidal people in living populations have higher diversity than healthy controls, specifically increased taxa associated with inflammation (Naseribafrouei et al., 2014). Therefore, there is a potential link between high microbial beta-dispersion and mental health that would be a promising area of future research. Previous work documented the association of postmortem microbiome diversity and other metrics to heart disease (Pechal et al., 2018). In the current research, cardiovascular disease had significantly higher beta-dispersion than any other type of death. Dysbiosis in the microbiomes of people with cardiovascular disease has been documented, as there may be a microbiome link to disease pathogenesis (Wilson Tang and Hazen, 2017). Based on our results, some deaths may benefit from microbial evidence more than others. Specifically, drug-related deaths, cardiovascular disease, and suicides prompt further investigation with the postmortem microbiome. It is important to note that other MOD/CODs may not preclude the decedent from having cardiovascular disease antemortem (i.e., a homicide victim could have cardiovascular disease), which could in turn affect the microbiome. In future studies, it would be pertinent to explore the interaction between cardiovascular disease and other MODs/CODs.

We chose multinomial logistic regression as a simple model that is often used in clinical settings (de Jong et al., 2019). However, multinomial logistic regression has limitations, and biases toward classifying categories with larger sample sizes (de Jong et al., 2019); thus, future modeling approaches may provide improved predictive ability for forensic use. We achieved marginal improvement of classification in these models with random forest indicator taxa compared to models using the full microbial community data set. This result was not entirely unexpected, as random forest model error rates ranged from: 53.1–64.4% (Supplementary Table S15). Random forest indicators derived from random forest models with high error (>50% error rate) did not improve multinomial regression models classifying M/COD. This illustrates that beta-dispersion can be calculated in a variety of ways, which has downstream effects on distinguishing categories of interest. Therefore, an objective approach to selecting beta-dispersion calculation should be used, as outlined in this study.

Instead, binary logistic regression models were most effective at improving model success. The categories with the highest classification success also had the largest sample size (natural deaths/accidents; cardiovascular disease/drug-related deaths), and were highly correlated, as most natural deaths were cardiovascular disease (42/57) and most accidents were drug-related deaths (59/71). There was some overlap in pathology among cardiovascular disease deaths and drug-related deaths (Molina et al., 2020), showcasing how our best performing logistic regression models have potential applications in forensic death determination. While our case studies would benefit from further exploration with larger datasets, we provided strong evidence that other comparisons differentiating MOD, such as suicide vs. non-suicides, could also prove useful for forensic death determination. Additionally, future research efforts may involve novel approaches to model parameterization better informed by the specific M/COD and underlying case context and characteristics.

While not the first study to classify M/COD from microbial communities (Pechal et al., 2018; Kaszubinski et al., 2019; Zhang et al., 2019), this is the first study to compare random forest classification and logistic regression performance using beta-dispersion. MOD classification success with microbial community random forest indicators alone (Kaszubinski et al., 2019) were comparable to multinomial logistic regression models built with only beta-dispersion (∼40%). Inclusion of case demographic data improved multinomial logistic regression model, which was consistent with previous random forest regression model accuracy of ears and nose body site communities (>60%) (Zhang et al., 2019). A strength of the MLR approach, is that it does not depend on specific indicator postmortem microbial taxa, which can vary across studies (Pechal et al., 2018; Kaszubinski et al., 2019). Furthermore, we suggest that microbial community information, either taxon dependent (e.g., indicator taxa) or not (e.g., beta-dispersion), could be an additional piece of evidence in M/COD determination. We wanted to identify if demographic data were indicative of certain M/CODs (e.g., age significantly higher in natural deaths or for cardiovascular disease) and were useful to supplement beta-dispersion in downstream modeling. We chose a slightly less conservative p-value so that potentially important demographic data were not prematurely removed. By including demographic data into our models, successful classification was improved rather than using microbial data alone, which is something to consider in future death investigation.

## Conclusion

Microbial community metrics, such as beta-dispersion, have potential forensic use in contributing to classification of M/COD during death investigation. This reflection is due to the antemortem link to the postmortem microbiome. We showed beta-dispersion increased based on disease status (cardiovascular disease) according to AKP, and beta-dispersion reflected M/COD, especially for cardiovascular disease and drug related deaths. While random forest is a useful tool for these types of datasets, MLR with beta-dispersion produced comparable results without reliance on specific microbial indicator taxa. Furthermore, we demonstrated circumstances where beta-dispersion could be used to distinguish MOD using two case studies; however, low and uneven sample size was an issue for all case studies. Despite the reduced power of these case studies, this workflow may be useful for other forensic practitioners to test within their own sample set, that encompass new locations and demographic data, to strengthen the antemortem link to the postmortem microbiome. As sample sizes increase for postmortem microbial studies, it may be necessary for large databases, or geographically and demographically specific data to train models with high success rates for practical use in forensic contexts. The methods outlined in this study serve as a guide to developing non-taxonomic indicator microbiome tools for other researchers and medical examiners in different geographic locations and investigation contexts. Ultimately, modeling beta-dispersion with case demographic data is a potential tool that could be useful for medical examiners during death investigation to combine with other methods of M/COD determination. The future of using postmortem microbiomes in forensic sciences continues to show promise.

## Data Availability Statement

Sequence data were archived through the European Bioinformatics Institute European Nucleotide Archive (www.ebi.ac.uk/ena) under accession number: PRJEB22642. The microbial community analyses are available as R code on GitHub (https://github.com/sierrakasz/AKP-betadisp-paper).

## Author Contributions

SK performed the data analysis and wrote the manuscript. JP, CS, HJ, and MB acquired the data. JP and KS helped design the case studies. JP, MM, and MB critically revised intellectual content. SK, JP, KS, CS, HJ, MM, and MB edited the manuscript and approved the final version. All authors contributed to the article and approved the submitted version.

## Conflict of Interest

The authors declare that the research was conducted in the absence of any commercial or financial relationships that could be construed as a potential conflict of interest.
