# Supplementary material for: Dysbiosis in the Dead: Human Postmortem Microbiome Beta-Dispersion as an Indicator of Manner and Cause of Death
Source: Front Microbiol. 2020 Sep 4;11:555347. doi: 10.3389/fmicb.2020.555347 (PMC7500141; doi:10.3389/fmicb.2020.555347)
Supplement: Supplementary file 1 [file Image_1.pdf]

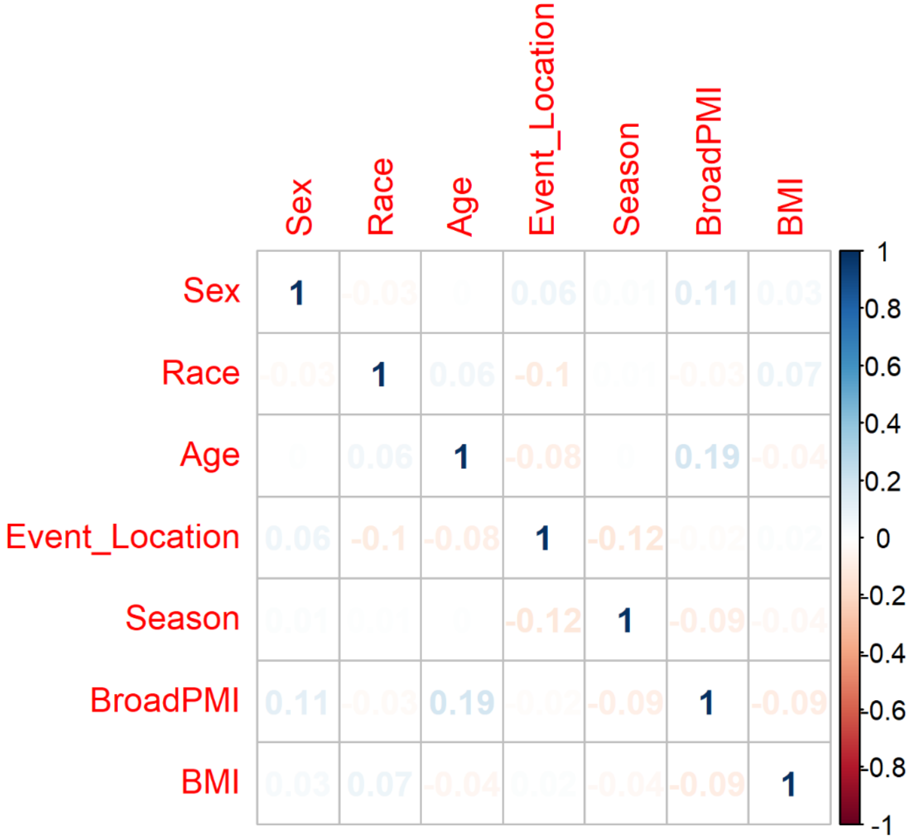

Supplemental Figure 1—Multicollinearity among metadata covariates. The x and y-axis correspond to the covariates. Correlation strength is noted in the boxes, corresponding to the covariate pairwise comparison. Color indicates positive or negative correlation.

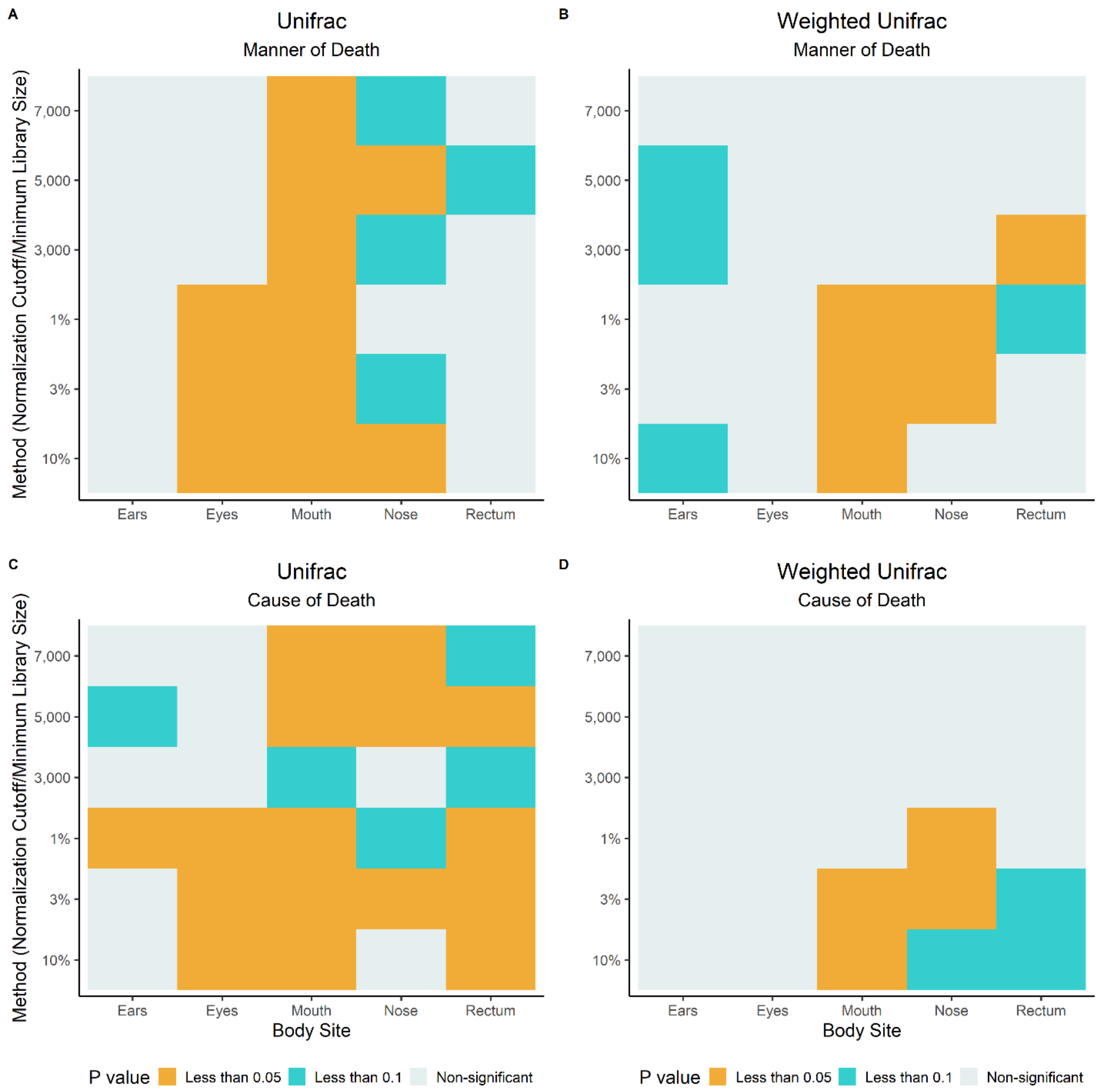

Supplemental Figure 2—Summary of Kruskal-Wallis results for normalization strategies determining beta-dispersion. Each body site is represented across the x-axes. Each level for the corresponding normalization strategy (sample percentage cutoff and minimum library sizes) are on the y-axes. Each box indicates the results of a Kruskal-Wallis test for either manner of death (A and B) or cause of death (C and D). Left-hand side of the figure includes Unifrac distances (A and C), while the right-hand side includes Weighted Unifrac distances (B and D). Significant (p value < 0.05) Kruskal-Wallis tests are indicated by gold, while nearly significant (p <0.1) are indicated by blue.

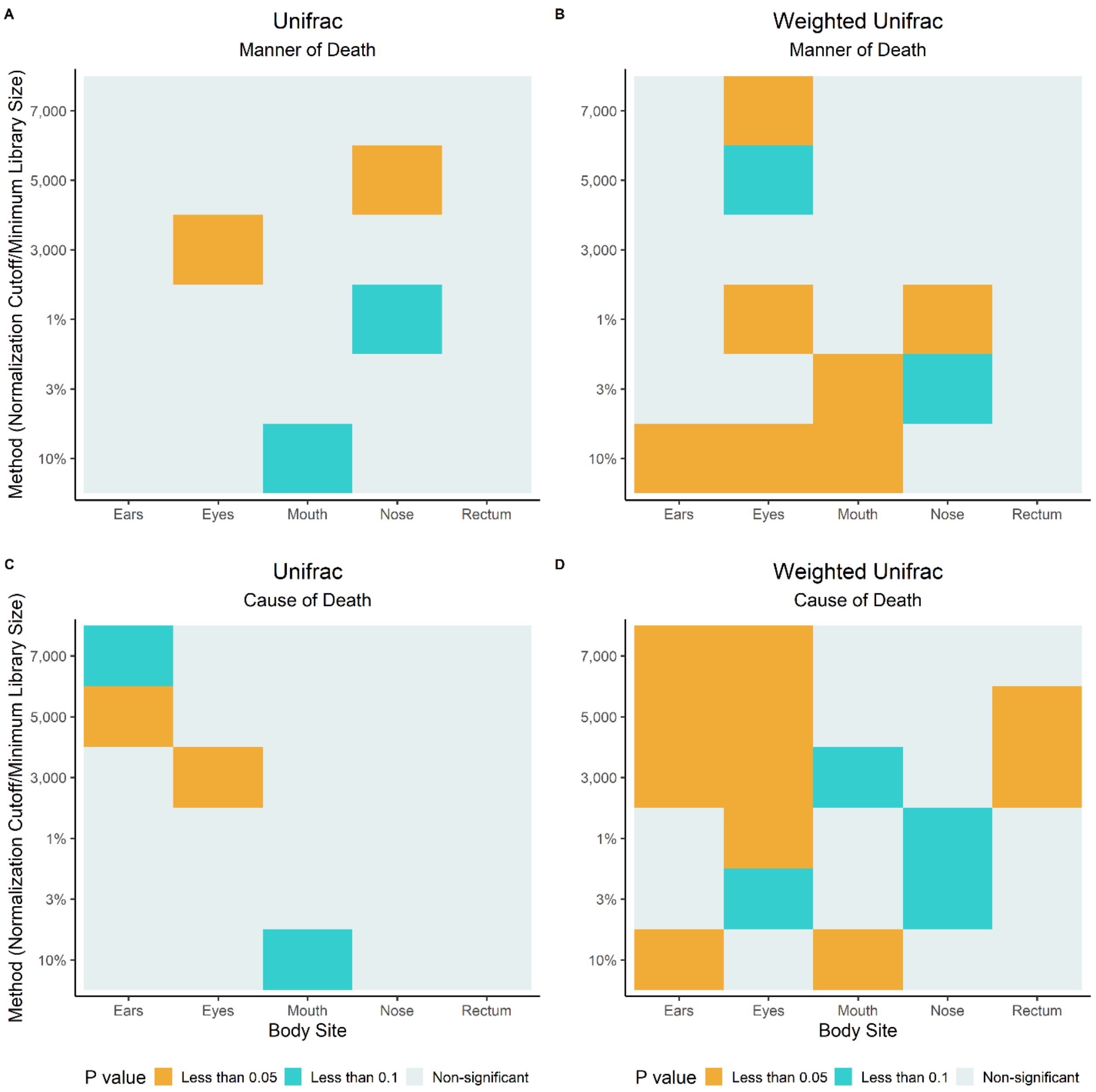

Supplemental Figure 3—Summary of Fligner-Killeen results for normalization strategies determining beta-dispersion. Each body site is represented across the x-axes. Each level for the corresponding normalization strategy (sample percentage cutoff and minimum library sizes) are on the y-axes. Each box indicates the results of a Fligner-Killeen test for either manner of death (A and B) or cause of death (C and D). Left-hand side of the figure includes Unifrac distances (A and C), while the right-hand side includes Weighted Unifrac distances (B and D). Significant (p value < 0.05) Fligner-Killeen tests are indicated by gold, while nearly significant (p <0.1) are indicated by blue.

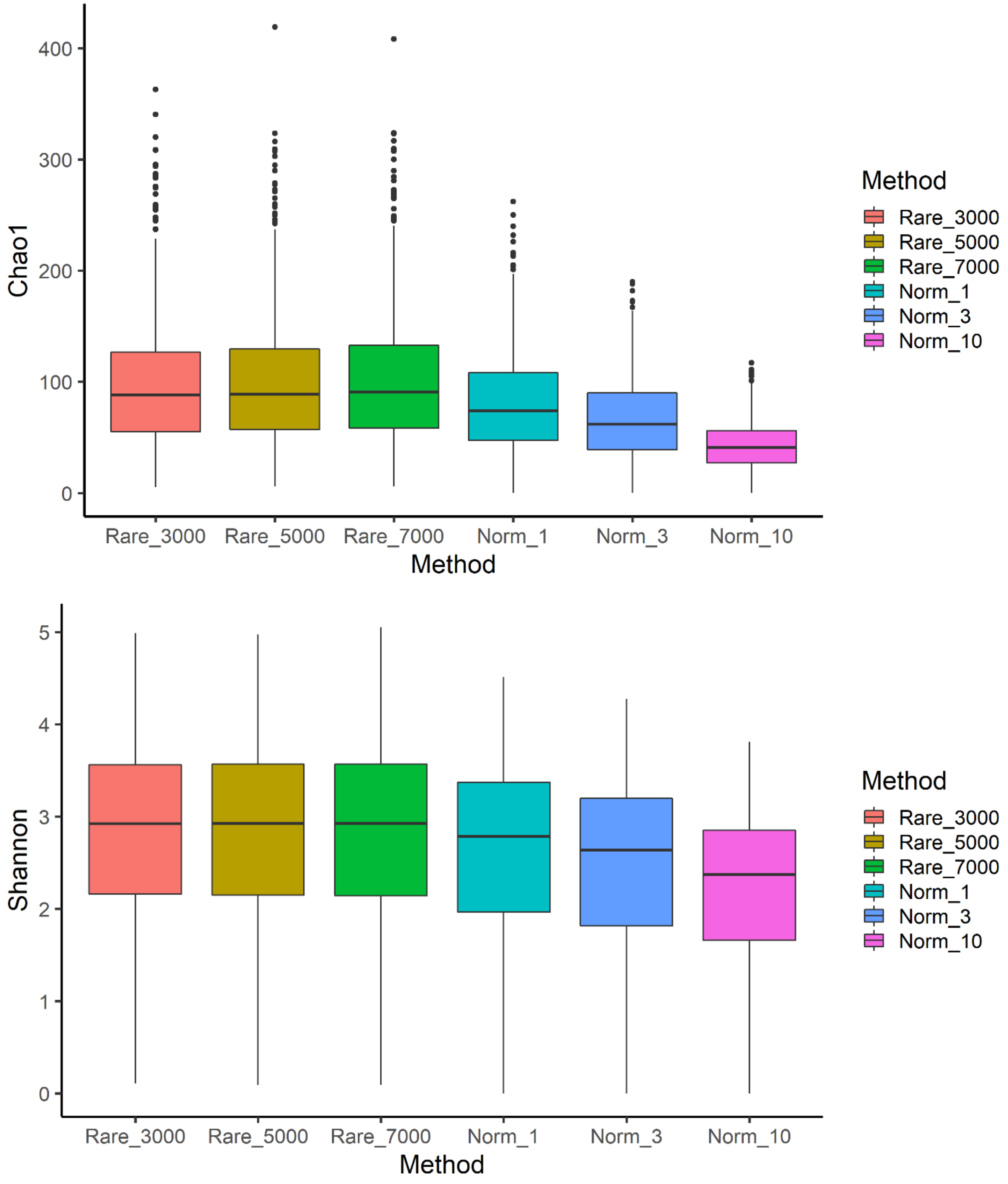

Supplemental Figure 4—Alpha-diversity metrics across normalization strategies. Chao1 (richness and Shannon diversity (richness and evenness) are reported. A significant decrease in alpha-diversity was found among percent cutoffs, and compared to minimum library sizes (Kruskal-Wallis and *post hoc* Nemenyi p value < 0.05).

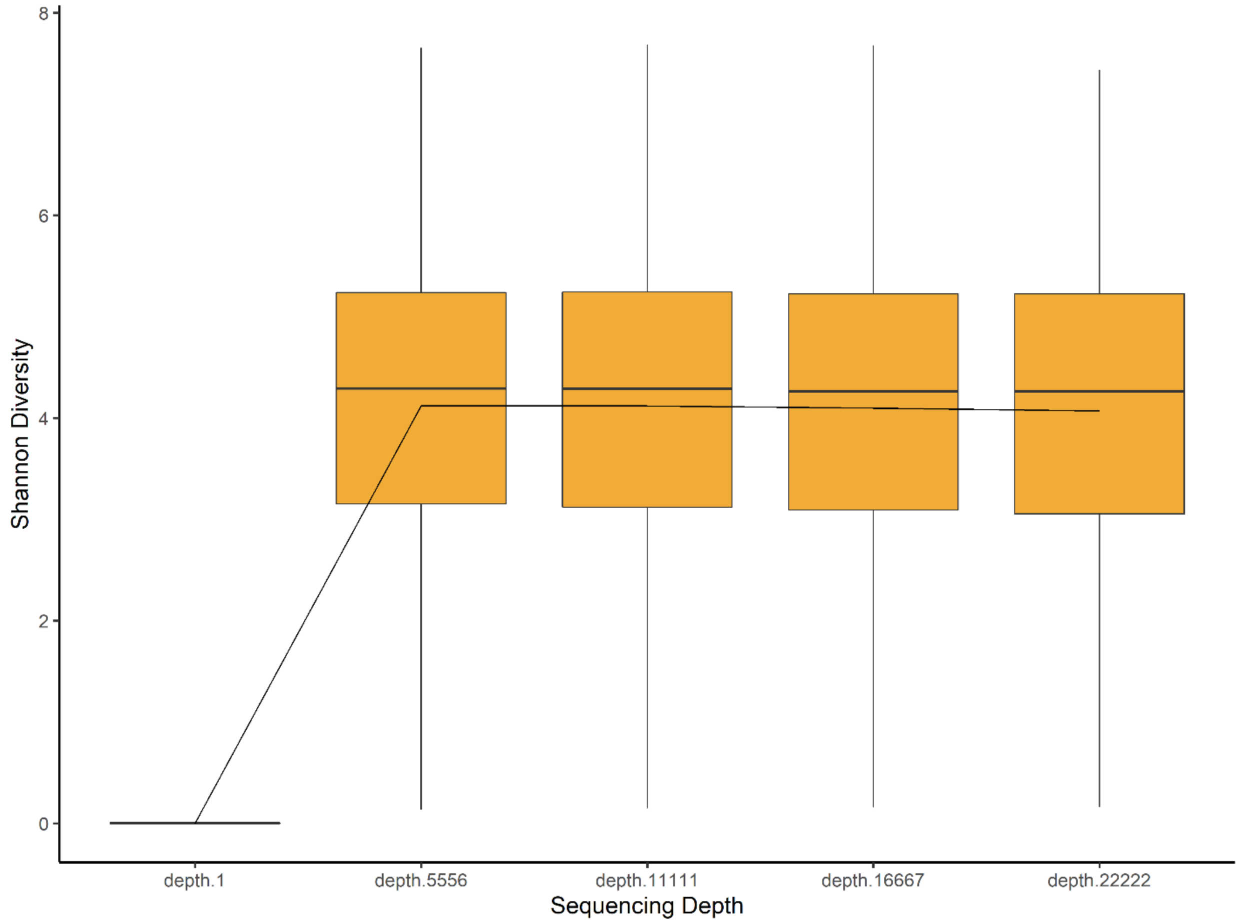

Supplemental Figure 5—Shannon diversity across sequencing depth among microbial samples.

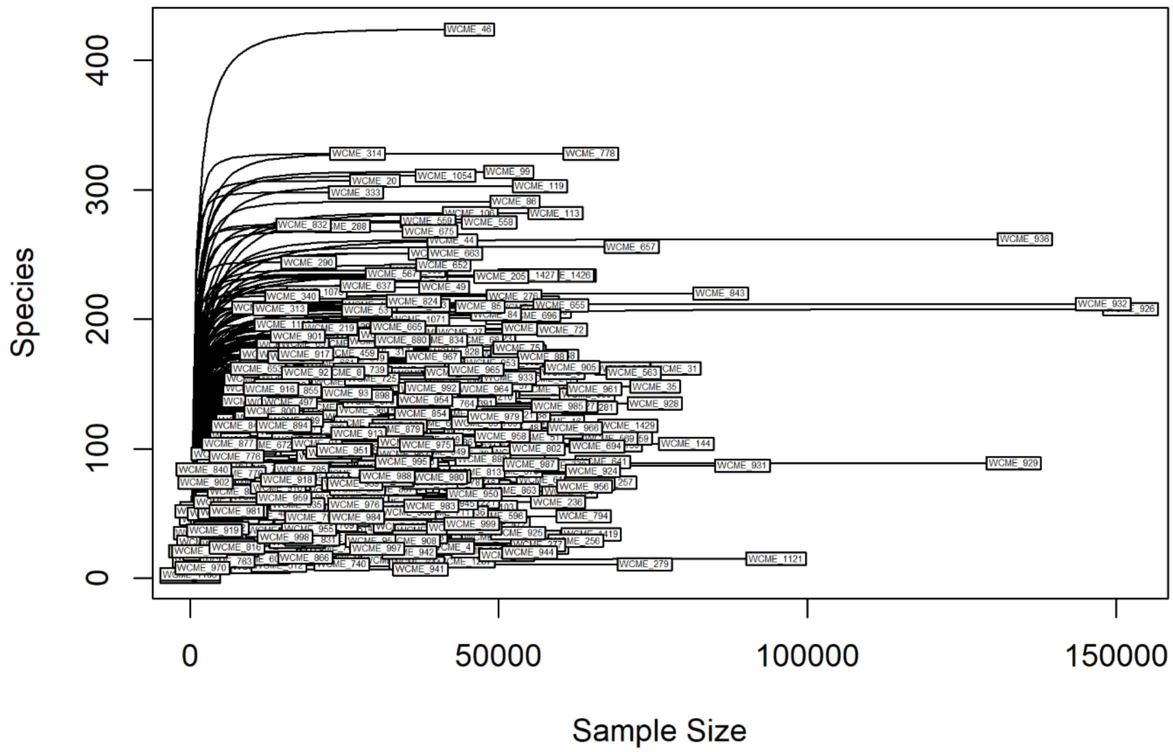

Supplemental Figure 6—Rarefaction curve among samples. The x-axis corresponds to the library size, or number of sequences per sample. The y-axis corresponds to the number of amplicon sequencing variants (ASV) per sample.
